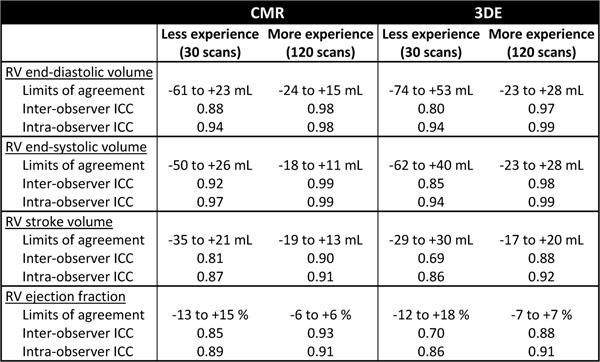# Learning curve for quantification of right ventricular size and systolic function in pulmonary arterial hypertension: comparison of cardiac magnetic resonance and three-dimensional echocardiography

**DOI:** 10.1186/1532-429X-14-S1-P81

**Published:** 2012-02-01

**Authors:** Jonathan Afilalo, Julia Grapsa, Giuliana Durighel, Declan ORegan, David Dawson, Robert A Levine, Petros Nihoyannopoulos

**Affiliations:** 1 Cardiac MRI, Beth Israel Deaconess Medical Center, Harvard University, Boston, MA, USA; 2 Echocardiography, Massachusetts General Hospital, Boston, MA, USA; 3 Cardiology, Hammersmith Hospital, Imperial College NHS Trust, London, UK

## Background

Quantification of right ventricular (RV) volumes is challenging, particularly in patients with pulmonary arterial hypertension (PAH) who often have dilated and dysfunctional RVs. Several studies have shown that cardiac magnetic resonance (CMR) and 3-dimensional echocardiography (3DE) provide very reliable measurements of RV volumes when performed by experienced observers; however, little is known about the reliability and learning curve of these measurements when performed by novice observers. We sought to measure the inter- and intra-observer reliability of RV volume quantification by CMR and 3DE as it pertains to a novice observer with a progressive amount of experience over a 2 year period.

## Methods

A prospective cohort of patients with idiopathic PAH was assembled at the Hammersmith Hospital (London, UK). Patients underwent CMR and 3DE at four time points: baseline, 6 months, 12 months, and 24 months. The scans were interpreted by a novice observer (level 1 training in echocardiography with no experience in CMR or 3DE) on the day of the scan, by the same novice observer in the week following, and by an expert observer (level 3 training with >5 years experience in CMR and 3DE). For CMR, SSFP short-axis cine images were acquired using a 1.5T system and endocardial contours were manually traced to obtain the RV end-diastolic volume, end-systolic volume, stroke volume, and ejection fraction. For 3DE, apical full-volume cine images were acquired and processed using a semi-automated algorithm (TomTec, Germany). Inter-observer reliability was calculated at the four time points with the use of the intraclass coefficient (ICC) and also the Bland-Altman limits of agreement; intra-observer variability was calculated with the ICC.

## Results

Thirty patients were enrolled, representing 120 CMR scans and 120 3DE scans. The mean baseline RV end-diastolic volume was 208 +/- 52 mL, RV end-systolic volume was 141 +/- 50 mL, and RV ejection fraction was 33 +/- 12 % (by CMR). At the time point with least experience (30 scans interpreted), the novice observer had modest agreement with the expert observer for RV measurements as evidenced by relatively wide limits of agreement (Table). At the time point with most experience (120 scans interpreted), the novice observer improved and had good agreement with the expert observer for all measurements. Although the level of agreement was comparable between CMR and 3DE after 120 scans, it took 60 scans for the novice observer to reach this level with CMR while it took 120 scans with 3DE. With regard to the inter-observer reliability, the novice observer had good reliability with both CMR and 3DE after the first 30 scans, improving to very good reliability after 120 scans; it took 60 scans to see this improvement with CMR while it took 120 scans to see it with 3DE.

## Conclusions

Novice observers can achieve high levels of inter- and intra-observer reliability for quantification of RV volumes after interpreting 60 CMR scans and 120 3DE scans.

## Funding

None.

**Figure 1:**